# Strengthening One Health: Global Applications of the Joint Risk Assessment Operational Tool

**DOI:** 10.3390/pathogens15080861

**Published:** 2026-08-19

**Authors:** Ong-orn Prasarnphanich, Sithar Dorjee, Rukshanda Ahmad, Richard Brown, Sharon Calvin, Hien Do, Peter Sousa Hoejskov, Gunel Ismayilova, Masaya Kato, Olena Kuriata, Jessica Kayamori Lopes, Heba Mahrous, Lisa Scheuermann, Tieble Traore, Linda Vrbova, Jan Trumble Waddell, Chadia Wannous, Endang Widuri Wulandari, Gyanendra Gongal, Stephane de la Rocque

**Affiliations:** 1 World Health Organization, Headquarters, 1211 Geneva, Switzerland; scheuermannl@who.int (L.S.); delarocques@who.int (S.d.l.R.); 2 World Health Organization, Regional Office for South-East Asia, New Delhi 110002, India; dorjees@who.int (S.D.); katom@who.int (M.K.); gongalg@who.int (G.G.); 3 Public Health Agency of Canada, Ottawa, ON K1A OK9, Canada; rukshanda.ahmad@phac-aspc.gc.ca (R.A.); sharon.calvin@phac-aspc.gc.ca (S.C.); linda.vrbova@phac-aspc.gc.ca (L.V.); jan.trumblewaddell@phac-aspc.gc.ca (J.T.W.); 4 World Health Organization, Country Office, Nonthaburi 11000, Thailand; drribr@gmail.com; 5 World Health Organization, Country Office, Hanoi 11119, Vietnam; doh@who.int; 6 World Health Organization, Regional Office for Europe, 2100 Copenhagen, Denmark; hoejskovp@who.int; 7 Food and Agriculture Organization of the United Nations, 00153 Rome, Italy; gunel.ismayilova@fao.org; 8 World Health Organization, Country Office, 04071 Kyiv, Ukraine; olena.kuriata@gmail.com; 9 World Health Organization, Regional Office for Western Pacific, Manila 1000, Philippines; kayamorij@who.int; 10 World Health Organization, Regional Office for Eastern Mediterranean, Cairo 11371, Egypt; hmahrous@who.int; 11 World Health Organization, Regional Office for Africa, Dakar 12500, Senegal; traoret@who.int; 12 World Organisation for Animal Health, 75017 Paris, France; c.wannous@woah.org; 13 World Health Organization, Country Office, Jakarta 12940, Indonesia; wulandarie@who.int

**Keywords:** surveillance, joint risk assessment, One Health, zoonotic diseases

## Abstract

Risk assessment is critical for managing health threats at the human–animal–environment interface, yet sector-specific approaches can result in fragmented actions. To address this gap, the Joint Risk Assessment Operational Tool (JRA OT), an operational tool of the Tripartite Zoonoses Guide, was developed by the Food and Agriculture Organization of the United Nations, the World Health Organization, and the World Organization for Animal Health. The JRA OT provides a structured framework for joint qualitative risk assessments that integrate multisectoral expertise to identify risk pathways, assess likelihood and impact, and develop consensus-based risk management and communication options. Surveillance systems play a critical role in this process by providing the multisectoral data needed to inform risk assessments, while the JRA process helps identify information gaps and guide the strengthening of integrated One Health surveillance. Implemented in at least 52 countries, the JRA OT has informed national mandates in Indonesia, Viet Nam, and Tanzania, regional strategies in West Africa, and adaptations in Canada. Cascade training has been used to build subnational capacity to facilitate roll out. Sustained leadership commitment, multisectoral coordination, routine applications, local adaptation, and capacity building remain essential for global implementation.

## 1. Introduction

Risk assessment (RA) is a process of collecting, evaluating, and documenting information to estimate the level or risk within a defined timeframe and location. Typically conducted within a single sector, this process relies on the best available information at the time of assessment. Sector-specific RAs are essential for human health, animal health, and environmental sectors to manage risks effectively within each sector’s unique context, priorities, and mandates. However, these assessments depend heavily on the availability and integration of surveillance data, which are often fragmented across sectors. This limits the effectiveness of both surveillance and risk assessment at the human–animal–environment interface or results in conflicting interventions among sectors.

Recognizing the need for coordinated, multidisciplinary efforts in managing zoonotic diseases, the Tripartite Zoonoses Guide (TZG) was co-developed and published in 2019 by the Food and Agriculture Organization of the United Nations (FAO), the World Health Organization (WHO), and the World Organization for Animal Health (WOAH) [1]. TZG provides the best practices for countries in adopting a multisectoral One Health approach to zoonotic disease management. It is supported by operational tools, including the Joint Risk Assessment operational tool (JRA OT) [2], to facilitate the implementation of this guidance.

This article presents a global implementation report on the adaptation, institutionalization, and operationalization of the JRA OT across countries and regions, underscoring the flexibility in adapting the approach to various contexts.

## 2. Materials and Methods

The JRA OT was officially launched in 2020 by the Tripartite (FAO, WHO, and WOAH). It offers a framework for joint, cross-sectoral qualitative risk assessment of zoonotic diseases, integrating expertise from relevant sectors, often ministries responsible for human health, animal health, and the environment. This approach enables risk management and communication options that reflect the complexities of health threats at the human–animal–environment interface. Importantly, the JRA OT complements—rather than replaces—sector-specific risk assessments. Sector-specific and joint risk assessments work in synergy. Various international organizations established sectoral risk assessment frameworks tailored to specific needs, such as rapid risk assessment of acute public health events (WHO) [3], import risk analysis (WOAH Terrestrial Animal Health Code) [4], pest risk assessment (International Plant Protection Convention) [5], and food safety risk analysis (Codex Alimentarius Commission) [6]. Annex S1 of the Supplemental Materials provides a table summarizing these frameworks.

The JRA process begins with establishing a steering committee, a lead, a technical team, and stakeholder groups. The steering committee defines the hazard(s), scope, purpose, and key objectives—a step known as risk framing. The lead manages the overall process, ensures communication, and guides the technical team in conducting assessments. Technical steps include identifying risk pathways, formulating assessment questions, and characterizing risks. Risk characterization involves reviewing data and multisectoral perspectives, identifying information gaps, estimating likelihood and impact, assigning uncertainties, and plotting estimates in a risk matrix. The outcomes are targeted, evidence-based, and consensus-driven risk management and communication options. Uncertainties and knowledge gaps identified during the JRA process can inform improvements in surveillance design, prioritization, and data sharing across sectors.

Information on JRA OT implementation, including the country and date of operationalization, was obtained from the International Health Regulations (IHR) Country Capacity Assessment, Monitoring, Evaluation and Planning Update, a weekly publication (the publication is distributed through an email distribution list; for more information, contact ihrmonitoring@who.int) compiled from reports submitted by WHO Headquarters and Regional Office focal points. Additional information was extracted from publicly available reports or provided by WHO staff at Headquarters, Regional, and Country Office levels who participated in the relevant JRA OT activities or received implementation updates from national counterparts.

## 3. Results

**Figure 1:**
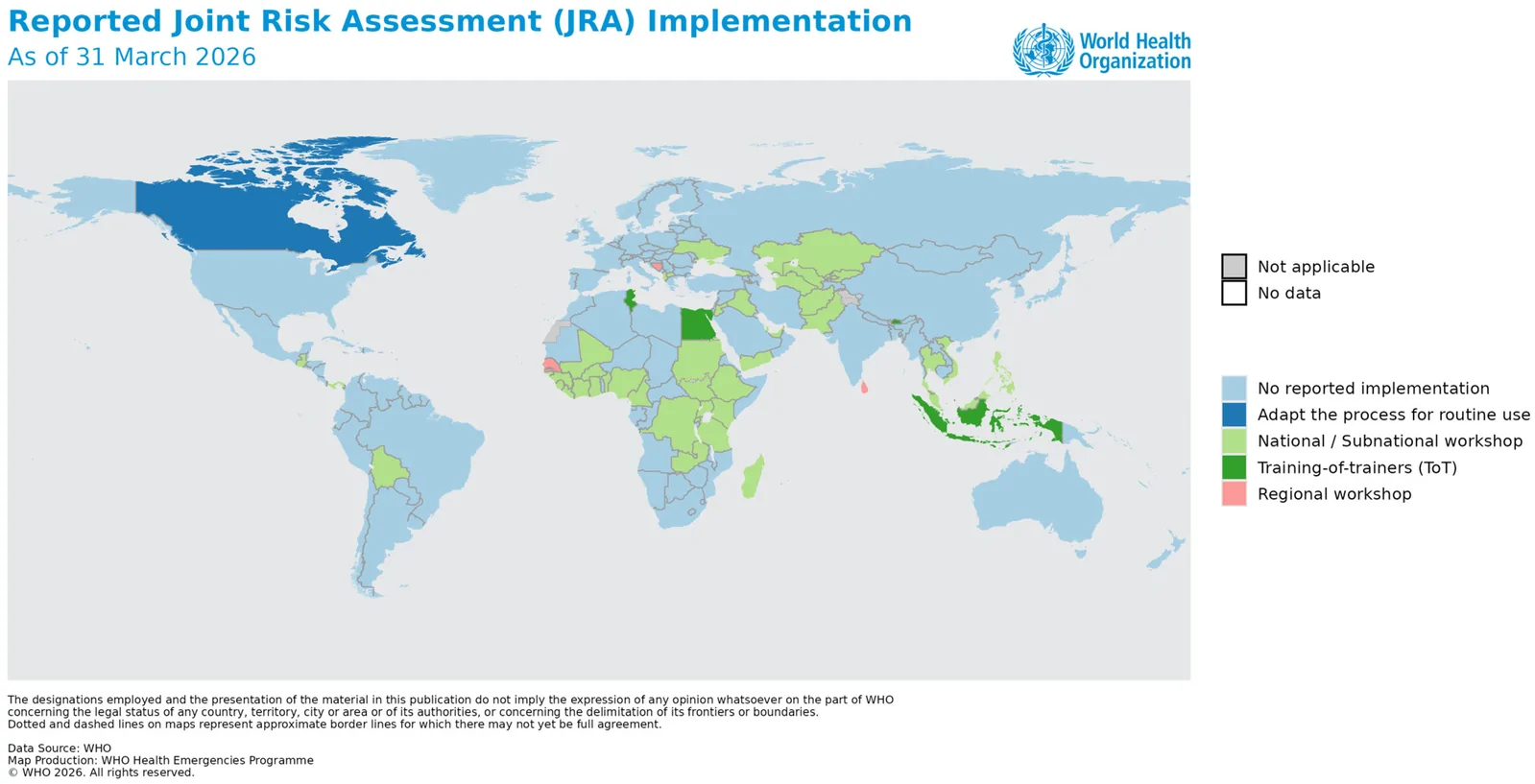
Reported Joint Risk Assessment (JRA) implementation as of 31 March 2026.

As of 31 March 2026, the implementation of the JRA OT was reported across 52 countries (Figure 1 and Annex S2 of the Supplemental Materials). These activities include the adaptation for routine use, training-of-trainers, and workshops held at regional, national, and subnational levels.

For the purposes of this article, adaptation of the JRA OT process was defined as the modification of the JRA methodology by a country or institution to align with its operational needs for routine use. A JRA workshop was defined as a structured workshop that applies the JRA OT methodology to guide participants through the steps of the joint risk assessment process. Prior to the tool’s official launch in 2020, pilot workshops were conducted to test the methodology and gather feedback for refinement and improvement. Following its launch, workshops may be conducted either to undertake an actual joint risk assessment of priority hazards or as an exercise designed to build participants’ knowledge and practical skills, enabling them to replicate the process independently when needed. Training-of-trainers (ToT) was defined as training provided to individuals expected to facilitate, lead, and support the operationalization of the JRA OT process within their respective countries. These activities can be implemented at different administrative levels, including regional (multicountry), national (country), and subnational (state, provincial, or district) levels.

A total of 16 pilot workshops were conducted during 2018–2019. Between 1 January 2020 and 31 March 2026, seventy-four JRA workshops were conducted, including 4 regional-level, 45 national-level, and 21 subnational-level workshops. During the same period, six training-of-trainers (ToT) were conducted. In addition to reporting the number of completed activities, countries’ experiences in implementing these activities and institutionalizing the JRA process are described below.

In Figure 1, countries indicated as “National/Subnational workshop” organized workshops either at the national level only or at both the national and subnational levels. Countries that adapted the process for routine use or held regional workshops did not report organizing national or subnational workshops. Similarly, countries classified as having conducted training-of-trainers (ToT)—except for Indonesia—did not report organizing national or subnational workshops. Indonesia was the only country that conducted ToTs and organized workshops at the national and subnational levels; however, for the purposes of the map, it is categorized under “Training-of-trainers (ToT).”

### 3.1. Flexibility in the JRA OT Implementation

The JRA OT can be applied for both preparedness planning and response to health events. Several countries conduct zoonotic disease prioritization before the JRA process to focus planning on the most critical threats. Examples include Bhutan, Jordan, North Macedonia, Ukraine, Viet Nam, and Zambia [7]. Not all prioritized threats are typically assessed, often due to feasibility constraints or insufficient data. Ukraine identified ten priority zoonotic diseases; however, only tularemia, anthrax, rabies, and salmonellosis were jointly assessed in 2023, considering the potential impact of the ongoing war [8].

The JRA process may be initiated to respond to an outbreak, as demonstrated by its application to COVID-19 in Senegal, or to assess the risk of disease introduction. For example, the Philippines assessed the risk of Nipah virus introduction following outbreaks in neighboring countries [9]. Likewise, Indonesia conducted a JRA in early 2026 in response to Nipah virus outbreaks reported in India and Bangladesh.

Routine JRAs are recommended to address evolving risk dynamics. Surveillance data serves as a key trigger for initiating JRAs and updating risk assessments and associated interventions over time. In Indonesia, JRAs have been conducted at the subnational level to assess priority zoonotic diseases and respond to concerns arising from increasing disease incidence and outbreaks, including rabies in East Nusa Tenggara. Similarly, Viet Nam regularly applies JRAs to prioritized diseases, including avian influenza, rabies, and anthrax [7].

### 3.2. Institutionalization of the JRA OT Process

The Public Health Agency of Canada’s (PHAC) division on risk assessment has adapted the JRA OT for its infectious disease risk assessments. The methodology aligns with its One Health Approach to Risk Assessment framework [10]. PHAC does not utilize the workshop format; instead, key components of the JRA OT have become the basis for PHAC’s risk assessment processes across diverse risk questions with committees supporting assessments [11]. Assessments of avian Influenza A(H5N1), measles, and Oropouche are examples of this adapted method [12].

Additionally, PHAC has further adapted the JRA methodology to enhance the assessment of likelihood and impact by expanding the impact categories to apply to infectious diseases beyond zoonoses, and by employing the STEEP (social, technological, economic, environmental, population health and health system, political and regulatory) categories to capture a broad range of impacts [11].

Countries like Indonesia, Viet Nam, and Tanzania institutionalize the JRA process in national regulations, guidelines, or planning frameworks. In 2018, Indonesia piloted the JRA OT using scenarios involving highly pathogenic avian influenza A(H5N1), A(H9N2), leptospirosis, and rabies. Another JRA pilot in 2019 assessed the risk of rabies introduction to the historically rabies-free Lombok Island. The JRA OT has since been incorporated into zoonosis prevention and control training and translated into Bahasa. The Regulation of the Coordinating Minister for Human Development and Cultural Affairs of the Republic of Indonesia (Number 7 of 2022) on Guidelines for the Prevention and Control of Zoonoses and Emerging Infectious Diseases provides JRA the legal basis as the national risk assessment framework [13].

Similarly, Viet Nam piloted the JRA OT in 2019 and has since applied it to avian influenza A(H5N1), A(H5N6), A(H5N8), A(H9N2), swine influenza A(H1N1)v, rabies, anthrax, and Streptococcus suis. In 2023, Viet Nam developed a national guideline to facilitate JRA applications. At the time of writing, government approval and institutionalization is ongoing [7].

The United Republic of Tanzania’s National One Health Strategic Plan (2022–2027) incorporates the JRA under Strategy Pillar 2: Surveillance, Detection, Prevention, and Control [14]. The JRA process informed the 2024 update of the Economic Community of West African States’ avian influenza strategy [15]. Albania also applied JRA findings in 2025 to develop its multisectoral Zoonotic Disease Prevention, Preparedness and Response Plan [16].

### 3.3. Advocacy and Cascade Training for JRA OT Implementations

Endorsement and sustained commitment from senior leadership and the broader workforce enable routine implementation of the JRA process. Risk assessors, including subject matter experts, must also be prepared to collaborate beyond traditional mandates of their organizations. Various models have been employed to build this capacity. These include training-of-trainers and using regional, national, or subnational JRA workshops as training opportunities.

A regional workshop organized by FAO, WOAH, and WHO was held in Sri Lanka, in 2023, bringing together representatives from Bangladesh, Bhutan, India, Indonesia, Maldives, Nepal, Sri Lanka, Thailand, and Timor-Leste [17]. Another multi-country workshop was conducted for the Western Balkans (Albania, Bosnia & Herzegovina, Kosovo (all references to Kosovo should be understood to be in the context of United Nations Security Council resolution 1244 (1999)), Montenegro, North Macedonia, and Serbia) and Moldova in 2024 [18]. These regional meetings allowed participants to share experience and practice using the JRA OT with scenarios of common zoonotic diseases.

Nigeria demonstrates effective cascade training and rollout of the JRA OT from national to state levels. The first National Operationalization Workshop took place in 2021, using rabies and Lassa fever as scenarios. Later that year, JRA training workshops were conducted for Kano, Kebbi, Enugu, and Rivers states, followed by Ebonyi, Kaduna, and Jigawa states in 2024. States were selected based on the presence of multisectoral coordination mechanisms at the subnational level [7]. Uzbekistan applied a similar approach where a national JRA workshop took place in January 2025, and sub-national training workshops followed in September and October 2025 [19].

## 4. Discussion

Since the first pilot in 2018, we observed common success factors and challenges in rolling out and institutionalizing the process in countries. Leadership buy-in across government stakeholders was a critical enabler for initiating and sustaining JRA OT process implementation. Engaging local administrations and the environment sector proved essential for comprehensive risk assessment at the human–animal–environment interface, while technical expertise from FAO, WHO, WOAH, and United Nations Environment Programme was highly valued. Incorporating the JRA OT process into mandates, regulations, and national planning further supports institutionalization, although it often requires time, as shown by examples from Indonesia and Viet Nam. Countries with existing multisectoral coordination mechanisms such as Nigeria and Thailand found it easier to convene stakeholders and maintain follow-up. Conversely, the JRA OT process itself can foster collaboration where such mechanisms could improve—a challenge that persists due to competing priorities and limited resources.

A major challenge identified was limited and fragmented surveillance data across sectors, which increases uncertainty in risk assessment. Strengthening One Health surveillance, requiring coordination and sharing of surveillance data among human, animal, and environment sectors, is therefore critical to improving the quality and timeliness of JRA outputs. To address this, countries should aim for a balance between the level of detail required and the practicality of supporting planning and response. Additional recommendations include updating JRAs routinely to account for evolving risks, adapting or simplifying the process for local and rapid application, and investing in long-term capacity building through in-person training or online resources, such as the Tripartite JRA course available on OpenWHO [20]. Monitoring and evaluation of disease management and communication measures are also critical to ensuring their effectiveness and informing continuous improvement.

## 5. Conclusions

By providing a structured yet flexible approach, the JRA OT offers a valuable resource for operationalizing One Health strategies across regional, national, and subnational levels. Central to this contribution is the inherent interdependence between surveillance and joint risk assessment. Surveillance generates the data needed to assess risk, while the JRA OT process identifies information gaps that guide and strengthen surveillance systems within a One Health framework.

## Data Availability

All reported Joint Risk Assessment Operational Tool (JRA OT) activities are published on https://extranet.who.int/sph/jra (accessed on 26 July 2026).

## Supplementary Materials

The following supporting information can be downloaded at: https://www.mdpi.com/article/10.3390/pathogens15080861/s1. Annex S1: Comparative Summary of International Risk Assessment Tools for Human Health, Animal Health, Food Safety, and Plant Health [2,3,4,5,6]; Annex S2: Reported JRA OT implementation as of 31 March 2026.

## Abbreviations

The following abbreviations are used in this manuscript:

JRA OT	Joint Risk Assessment operational tool
TZG	Tripartite Zoonoses Guide
FAO	Food and Agriculture Organization of the United Nations
WHO	World Health Organization
WOAH	World Organization for Animal Health
