# Supplementary material for: Strengthening One Health: Global Applications of the Joint Risk Assessment Operational Tool
Source: Pathogens. 2026 Aug 19;15(8):861. doi: 10.3390/pathogens15080861 (PMC13516726; doi:10.3390/pathogens15080861)
Supplement: Supplementary file 1 [file pathogens-15-00861-s001.zip › pathogens-4362234-supplementary.pdf]

Annex S1: Comparative Summary of International Risk Assessment Tools for Human Health, Animal Health, Food Safety, and Plant Health

Risk assessment approach	One Health / Joint Risk Assessment (FAO, WHO, WOAH)	Public Health (WHO)	Animal Health (WOAH)	Food Safety (Codex)	Plant Health (IPPC)
**Name of RA tool or framework**	Joint risk assessment operational tool [1]	Rapid risk assessment of acute public health events [2]	Import risk analysis [3]	Risk Analysis Principles Applied by the Codex Committee on Food Additives and Contaminants [4]	Pest risk analysis [5]
**Steps involved**	10 steps process:<br>(1) Establish and convene a national steering committee.<br>(2) Identify a JRA lead.<br>(3) Establish and convene technical team.<br>(4) Establish and convene stakeholder groups.<br>(5) Risk framing<br>(6) Identify and diagram the risk pathway.<br>(7) Formulate and document risk assessment questions.<br>(8) Characterize the risk.<br>(9) Identify risk management options	(1) Event Detection.<br>(2) Risk assessment includes hazard assessment, exposure assessment, context assessment, and risk characterization.<br>(3) Control measures/risk management.<br>(4) Risk communication.<br>(5) Evaluation.	(1) Hazard identification.<br>(2) Risk Assessment – including release assessment, exposure assessment, consequence assessment, and risk estimation.<br>(3) Risk Management.<br>(4) Risk Communication.	(1) Risk Assessment – including hazard identification, exposure assessment, and hazard characterization.<br>(3) Risk Management.<br>(4) Risk Communication	(1) Initiation.<br>(2) Pest risk assessment – including pest categorization, assessment of the probability of introduction (entry, establishment, and spread), impact, overall assessment of risk, and uncertainty.<br>(3) Pest risk management.<br>(4) Documentation.

Risk assessment approach	One Health / Joint Risk Assessment (FAO, WHO, WOAH)	Public Health (WHO)	Animal Health (WOAH)	Food Safety (Codex)	Plant Health (IPPC)
	and communication messages.<br>(10) Document the assessment.				
**Focus**	Integrative approach assessing the health risks at the human-animal-environment interface to achieve optimal health outcomes.	Quickly evaluating the risk to public health due to infectious diseases or other health threats to inform immediate decision-making.	Evaluating the risk of importing animals or animal products that could introduce diseases to domestic populations.	Ensuring that food remains safe for consumption by assessing the risks posed by biological, chemical, and physical hazards in food products.	Identifying, assessing, and managing the risks posed by pests to crops, forests, and the natural environment.
**Methodology**	Multidisciplinary and multisectoral approach, combining data and risk assessment techniques from public health, animal health, plant health, and environmental science.	Rapid assessments are done in real-time, often in response to outbreaks or emergencies. These assessments prioritize speed and may rely on incomplete data to estimate the potential impact and spread of disease.	Utilizing qualitative and quantitative measures to assess the likelihood and potential impact of disease introduction through international trade.	Typically quantitative, focusing on the likelihood of exposure to hazards and the severity of health outcomes. This approach often uses data on hazard prevalence, concentration levels, and consumption patterns.	Including pest risk identification, assessment, and management. It often involves evaluating the probability of pest entry, establishment, and spread, as well as the magnitude of potential economic and environmental impacts.
**Key components**	Collaboration among sectors to identify shared risks and co-benefits, assessing how interventions in one sector affect others, and	Identification of the agent or hazard, modes of transmission, affected populations, and potential for spread; often includes	Identification of pathogens, assessment of the exporting country's disease status, the susceptibility of local species, and the	Hazard identification, hazard characterization (toxicology and dose-response), exposure assessment, and risk characterization.	Pest identification, pathway analysis, climatic and ecological assessments to determine establishment

Risk assessment approach	One Health / Joint Risk Assessment (FAO, WHO, WOAH)	Public Health (WHO)	Animal Health (WOAH)	Food Safety (Codex)	Plant Health (IPPC)
	developing strategies that mitigate risks.	recommendations for public health actions.	potential for disease spread.		potential, and evaluation of control measures.
**Main results**	Science-based risk management and risk communication measures that are aligned between sectors.	Documentation of the process assessing acute public health events including:<br>- Which risk and control measures were assessed<br>- The methods used to assess them<br>- Why they were considered important<br>- Their order of priority.	Estimates of risks associated with the importation of animals, animal products, animal genetic material, feedstuffs, biological products and pathological material.	Food safety risk estimates presented in a readily understandable and made available to other risk assessors and interested parties.	Categorization of pest risks (acceptable vs. not acceptable) and corresponding management options.

References

1. World Health Organization, Food and Agriculture Organization of the United Nations, World Organisation for Animal Health. *Joint Risk Assessment Operational Tool (JRA OT): An Operational Tool of the Tripartite Zoonoses Guide*; WHO, FAO, WOAH: Geneva, Switzerland, 2021. Available online: https://www.who.int/publications/i/item/9789240015142 (accessed on 22 January 2026).
2. World Health Organization. Rapid Risk Assessment of Acute Public Health Events. 1 January 2012. Available online: https://www.who.int/publications/i/item/rapid-risk-assessment-of-acute-public-health-events (accessed on 11 March 2026).
3. World Organisation for Animal Health (WOAH). Chapter 2.1: Import Risk Analysis. 2018. Available online: https://www.woah.org/fileadmin/Home/eng/Health_standards/tahc/2018/en_chapitre_import_risk_analysis.htm (accessed on 11 March 2026).
4. International Plant Protection Convention (IPPC). Framework for Pest Risk Analysis. 28 July 2025. Available online: https://www.ippc.int/en/publications/framework-pest-risk-analysis/ (accessed on 11 March 2026).

5. Food and Agriculture Organization of the United Nations (FAO). Codex Alimentarius Commission—Procedural Manual. Section III: Working Principles for Risk Analysis. Available online: https://www.fao.org/4/a0247e/a0247e04.htm (accessed on 11 March 2026).

Supplemental Materials to the article titled ***Strengthening One Health: Global Applications of the Joint Risk Assessment Operational Tool***

Annex S2. Reported JRA OT implementation as of 31 March 2026

Year	Country / region	Date of workshop
**2026 (2 workshops)**	Egypt	16-17 February
	Indonesia	12 February-17 March
**2025 (11 workshops)**	Uzbekistan	22-24 January
	Albania	13-15 May
	North Macedonia	21-23 May
	Kyrgyzstan	3-5 June
	Viet Nam (subnational for central region)	18 July
	Viet Nam (subnational for southern region)	21-22 August
	Viet Nam (subnational for central region)	24 October
	Uganda	10-14 November
	Yemen	17-21 November
	Turkmenistan	26-28 November
	Viet Nam (subnational for south-central region)	12-13 December
**2024 (14 workshops)**	Bolivia	2-4 April
	Viet Nam (subnational for central region)	4-6 April
	Liberia	9-13 April
	Guatemala	23-24 April
	Bhutan (Training-of-trainers)	2-4 May
	Malaysia	12-15 June
	Madagascar	23-25 July
	Nigeria (subnational for Kaduna, Jigawa and Ebonyi States)	26-28 August
	Mali	27-30 August
	Western Balkans sub-regional workshop (for Albania, Bosnia & Herzegovina, Kosovo[1], Moldova, Montenegro, North Macedonia, and Serbia), held in Bosnia & Herzegovina	11-13 September
	Indonesia (subnational for East Java)	17-20 September
	West Africa sub-regional (for Guinea, Guinea Bissau, The Gambia and Senegal), held in Senegal	1-3 October
	Kazakhstan	12-14 November
	Zambia	18-22 November
**2023 (15 workshops)**	Indonesia (subnational South Kalimantan)	13-15 March
	Regional workshop for West Africa, held in Senegal	29-31 March
	Democratic Republic of Congo	17-20 April
	Guinea (subnational for Mamou)	11-14 May

[1] All references to Kosovo should be understood to be in the context of United Nations Security Council resolution 1244 (1999).

Supplemental Materials to the article titled ***Strengthening One Health: Global Applications of the Joint Risk Assessment Operational Tool***

Year	Country / region	Date of workshop
	Guinea (subnational for N'Zérékoré)	17-20 May
	Regional workshop for WHO South-East Asia	25-27 July
	Indonesia (subnational for Timor island East Nusa Tenggara)	1-4 August
	Ukraine	29-31 August
	The Gambia	25-28 September
	Guinea	8-30 September
	Viet Nam	10-11 August
	Benin	20-24 November
	The Philippines	27-30 November
	Zambia	5-8 December
	Iraq	26-28 December
**2022 (10 workshops)**	Jordan	7-10 March
	Indonesia (subnational for East Java)	22-24 March
	Qatar	26-28 March
	Viet Nam<br>(subnational for northern and southern regions)	5-6 May
	United Arab Emirates	27-30 July
	Burkina Faso	22-26 August
	Indonesia (subnational for Demak)	12-14 September
	Indonesia (subnational for DKI Jakarta)	10-14 October
	Indonesia (subnational for Central Java)	17-21 October
	Indonesia (subnational for DKI Jakarta)	26 October
**2021 (15 workshops)**	Democratic Republic of Congo	19-22 January
	Nigeria	8- 10 March
	Ethiopia	2-6 March
	Thailand	23-26 March
	Liberia	29-31 March
	Cameroon	29-31 March
	South Sudan	3-7 May
	Kenya	2-4 June
	Sierra Leone	26-29 July
	Indonesia (subnational for DKI Jakarta)	3-9 August
	Nigeria (subnational for Kano, Kebbi, Enugu and Rivers states)	9-11 August
	Cameroon	16-20 August
	Sudan	28-31 August
	Senegal	September
	Guinea	23-26 November
**2020 (7 workshops)**	Tunisia (Training-of-trainers) for North African Countries	28-30 January

Supplemental Materials to the article titled ***Strengthening One Health: Global Applications of the Joint Risk Assessment Operational Tool***

Year	Country / region	Date of workshop
	Egypt (Training-of-trainers)	1-5 October
	Indonesia (Training-of-trainers)	3 batches of five-day training during 30 November – 18 December
	Tanzania (subnational for Zanzibar)	14-17 December
	Senegal	15-18 December
**2019 (15 pilots)**	Viet Nam	20-22 February
	Panama	25 February – 01 March
	Tanzania	25-29 March
	Indonesia (subnational for West Nusa Tenggara)	4-7 April
	Pakistan	15-18 April
	Senegal	7-10 May
	Georgia	2-5 July
	Ethiopia	17-19 July
	Ghana	23-26 July
	Rwanda	30 July – 2 Aug
	Kenya	13– 16 August
	Afghanistan	25-28 August
	Cote d'Ivoire	11-14 November
	Cameroon	19 – 22 November
	Uganda	2-6 December
**2018 (1 pilot)**	Indonesia	13-16 March
